# Effects of sex and DTNBP1 (dysbindin) null gene mutation on the developmental GluN2B-GluN2A switch in the mouse cortex and hippocampus

**DOI:** 10.1186/s11689-016-9148-7

**Published:** 2016-05-01

**Authors:** Duncan Sinclair, Joseph Cesare, Mary McMullen, Greg C Carlson, Chang-Gyu Hahn, Karin E Borgmann-Winter

**Affiliations:** Department of Psychiatry, Neuropsychiatric Signaling Program, University of Pennsylvania, Philadelphia, PA USA; Present address: Schizophrenia Research Laboratory, Neuroscience Research Australia, Randwick, New South Wales Australia; University of Pennsylvania, Philadelphia, PA USA; Department of Child and Adolescent Psychiatry, Children’s Hospital of Philadelphia, Philadelphia, PA USA

**Keywords:** Development, NMDA, GluN2B, Sex difference, Postsynaptic density, Phosphorylation, Cortex, Hippocampus, Dysbindin, DTNBP1

## Abstract

**Background:**

Neurodevelopmental disorders such as autism spectrum disorders and schizophrenia differentially impact males and females and are highly heritable. The ways in which sex and genetic vulnerability influence the pathogenesis of these disorders are not clearly understood. The *n*-methyl-d-aspartate (NMDA) receptor pathway has been implicated in schizophrenia and autism spectrum disorders and changes dramatically across postnatal development at the level of the GluN2B-GluN2A subunit “switch” (a shift from reliance on GluN2B-containing receptors to reliance on GluN2A-containing receptors). We investigated whether sex and genetic vulnerability (specifically, null mutation of DTNBP1 [dysbindin; a possible susceptibility gene for schizophrenia]) influence the developmental GluN2B-GluN2A switch.

**Methods:**

Subcellular fractionation to enrich for postsynaptic density (PSD), together with Western blotting and kinase assay, were used to investigate the GluN2B-GluN2A switch in the cortex and hippocampus of male and female DTNBP1 null mutant mice and their wild-type littermates. Main effects of sex and DTNBP1 genotype, and interactions with age, were assessed using factorial ANOVA.

**Results:**

Sex differences in the GluN2B-GluN2A switch emerged across development at the frontal cortical synapse, in parameters related to GluN2B. Males across genotypes displayed higher GluN2B:GluN2A and GluN2B:GluN1 ratios (*p* < 0.05 and *p* < 0.01, respectively), higher GluN2B phosphorylation at Y1472 (*p* < 0.01), and greater abundance of PLCγ (*p* < 0.01) and Fyn (*p* = 0.055) relative to females. In contrast, effects of DTNBP1 were evident exclusively in the hippocampus. The developmental trajectory of GluN2B was disrupted in DTNBP1 null mice (genotype × age interaction *p* < 0.05), which also displayed an increased synaptic GluN2A:GluN1 ratio (*p* < 0.05) and decreased PLCγ (*p* < 0.05) and Fyn (only in females; *p* < 0.0005) compared to wild-types.

**Conclusions:**

Sex and DTNBP1 mutation influence the GluN2B-GluN2A switch at the synapse in a brain-region-specific fashion involving pY1472-GluN2B, Fyn, and PLCγ. This highlights the possible mechanisms through which risk factors may mediate their effects on vulnerability to disorders of NMDA receptor dysfunction.

**Electronic supplementary material:**

The online version of this article (doi:10.1186/s11689-016-9148-7) contains supplementary material, which is available to authorized users.

## Background

The maturation of neural circuitry across postnatal development is a dynamic and multifaceted process, which is vital for healthy adult brain function and cognition. A well-characterized and important aspect of this developmental program is the change in the subunit composition of the *n*-methyl-d-aspartate (NMDA) receptor. The heteromeric NMDA receptor is composed of two GluN1 subunits and a combination of two other subunits, which in the cortex and hippocampus are predominantly either two GluN2A subunits, two GluN2B subunits, or one of each [1, 2]. The subunit composition of NMDA receptors influences their channel properties, such as calcium permeability [3–5] and open probability [6], and hence impacts their role in synaptic plasticity (for review, see [7]). Across postnatal development in rodents and humans, the relative expression of the GluN2B subunit of the NMDA receptor decreases, while the expression of GluN2A increases [2, 8–14]. As a result, the NMDA receptor becomes less sensitive to blockade by GluN2B antagonists ifenprodil, CP101,606, and Ro25-6981 [9, 15–18]. These changes are accompanied by concurrent changes in electrophysiological properties, such as increasing excitatory postsynaptic current (EPSC) amplitude and decreasing decay time [15, 17], and reflect the healthy, activity-dependent maturation of the brain [19–21].

NMDA receptor signaling dysregulation has been implicated in schizophrenia, autism spectrum disorder (ASD), epilepsy, and intellectual disability, which are considered disorders of brain development [22–26]. NMDA receptor antagonists ketamine and PCP can induce psychosis symptoms in healthy individuals and worsen such symptoms in individuals with schizophrenia [27–29]. Altered NMDA receptor signaling in the prefrontal cortex of individuals with schizophrenia has been identified using a paradigm in which intracellular signaling downstream to receptor activation was monitored [30, 31]. In addition, altered expression of NMDA receptor subunits was shown in the whole brain homogenates [32] and in the postsynaptic density (PSD) [33], a cellular micro-domain which is a hub for postsynaptic signaling events. Genetic variants in the GluN2B gene, GRIN2B, have been implicated in sporadic ASD [34–38], while mutations of GRIN2B and GRIN2A (the GluN2A gene) have been associated with epilepsy and intellectual disability [39–42]. NMDA receptor knockout mice exhibit a range of neurological and behavioral deficits relevant to both schizophrenia and ASD [43–47]. Given that NMDA receptor abnormalities are implicated in schizophrenia and ASD, it is plausible that developmental disruption of the GluN2B-GluN2A switch may play a role in the pathogenesis of these disorders.

If the developmental GluN2B-GluN2A switch does play a role in the emergence of schizophrenia and ASD, then this process may also be impacted by risk factors for these illnesses. Two key factors which influence risk for schizophrenia and ASD are sex [48–51] and genetic vulnerability [52–54]. Evidence that sex modifies risk for schizophrenia and ASD comes from epidemiological studies which reveal that both disorders are more common in males, particularly ASD [55–57]. On average, schizophrenia is diagnosed earlier in males than in females [48–51], around adolescence and young adulthood when sexual dimorphism in the brain increases [58]. Males also experience greater severity of some schizophrenia symptoms [59, 60] even prior to conversion to psychosis [61]. In ASD, some evidence supports the theory that the brain is excessively masculinized [62, 63]. Genetic vulnerability also impacts risk for highly heritable neurodevelopmental disorders such as schizophrenia [52–54] and ASD [64, 65], likely via the combined influence of multiple risk variants. Among the genes which may increase the susceptibility to schizophrenia and ASD is DTNBP1 (dysbindin). Dysbindin is developmentally regulated [66] and contains single nucleotide polymorphisms (SNPs) identified as possible schizophrenia risk variants in genetic association studies prior to the GWAS era [67–70]. Some of these DTNBP1 SNPs have been associated with more severe psychotic symptoms [71]. They may also contribute to differences in structural brain development and cognitive ability in the general population [72–74]. DTNBP1 is also contained within a region on chromosome 6 which has been linked to ASD [75, 76] and is regulated by MeCP2 [77], the gene whose mutation causes Rett syndrome [78]. DTNBP1 mRNA and protein expression are decreased in the hippocampus and dorsolateral prefrontal cortex of individuals with schizophrenia [79, 80], while the DTNBP1 promoter is hypermethylated in the saliva and brain in individuals with schizophrenia [81, 82]. DTNBP1 null mutant mice, which do not express dysbindin protein, display cellular and functional abnormalities in the brain of relevance to schizophrenia, such as decreased GRIN1 mRNA expression [83], increased surface GluN2A protein expression [84], altered prepulse inhibition [85, 86], decreased hippocampal long-term potentiation [84, 87], and impaired working memory [83, 85]. It is not known whether sex and DTNBP1 mutation impact the developmental GluN2B-GluN2A switch in the brain, thereby increasing risk for schizophrenia and/or ASD.

Therefore, in this study, we investigated the effects of sex and dysbindin null mutation on the GluN2B-GluN2A switch in the frontal cortex and hippocampus. Using female and male wild-type (WT), heterozygous DTNBP1 null mutant [DTNBP1(+/−)] and homozygous null mutant [DTNBP1(−/−)] mice aged 7, 14, 28, and 56 days, we focused on events at the synapse using a biochemical approach to enrich for PSD proteins. This PSD enrichment enabled targeted quantification of proteins at the synapse, where NMDA receptor signaling plays a key role in excitatory neurotransmission and cognition [88]. We hypothesized that sex and/or DTNBP1 null mutation would disrupt the normal patterns of synaptic expression of GluN2B and GluN2A subunits and associated proteins across postnatal development.

## Methods

### Mouse breeding

Male and female heterozygous dysbindin mutant mice on a C57BL/6 background were bred as previously described [86, 89]. Genotyping of offspring was performed by duplex PCR, using DNA from tail snips and published primer sequences which span the deleted segment of DTNBP1 [90]. Mice in P28 and P56 age groups were weaned at P21. Animals were group housed in standard housing throughout the study. After weaning, males and females were housed in the same room under the same conditions. All protocols for animal care and use were undertaken in accordance with the University Laboratory Animal Resources guidelines and approved by the University of Pennsylvania Institutional Animal Care and Use Committee (protocol # 803572).

### Tissue collection

Homozygous DTNBP1(−/−) null mutant and heterozygous DTNBP1(+/−) null mutant mice, along with their wild-type (WT) littermates, were euthanized at postnatal days 7 (P7 group), 14–15 (P14 group), 28–30 (P28 group), and 56–60 (P56 group) by cervical dislocation without anesthesia. The frontal cortex (all cortex rostral to the genu of the corpus callosum) and hippocampus tissues were dissected on ice and immediately frozen. From the frontal cortex, DTNBP1(−/−), DTNBP1(+/−), and WT tissues were used, while from the hippocampus, DTNBP1(−/−) and WT tissues were used.

### PSD enrichment

To prepare subcellular fractions enriched for the PSD (referred to as PSD enrichments henceforth), the well-characterized insolubility of the PSD, and proteins strongly bound to it, in Triton X-100 [91, 92] was leveraged using a protocol described elsewhere [93]. Frontal cortical or hippocampal mouse brain tissue (10–30 mg) for each sample was homogenized using a Teflon homogenizer in 25 mM Tris (pH 7.4), 0.32 M sucrose, 1 mM EDTA, 1 mM EDTA, 1 mM Na_3_VO_4_, 5 mM NaF with phosphatase inhibitor cocktail (Sigma-Aldrich, St. Louis, MO, USA), and protease inhibitor cocktails II and III (Sigma-Aldrich). The homogenate was centrifuged at 1000*g* for 20 min at 4 °C, after which the supernatant was collected and spun again at 16,000*g* for 30 min at 4 °C. The resultant crude synaptic membrane (cSM) pellet was washed once with, then resuspended in, 25 mM Tris, 1 mM EDTA, 1 mM EDTA, 1 mM Na_3_VO_4_, 5 mM NaF with phosphatase/protease inhibitors, and total protein quantified by Lowry assay. Of this cSM preparation, 20–80 μg for each sample was further diluted in 800 μl of 1 % Triton X-100 buffer (pH 6.0) containing 1 % Triton X-100, 10 mM Tris (pH 6.0), 1 mM EDTA, 1 mM EGTA, 100 μM Na_3_VO_4_, 1 mM NaF, and phosphatase/protease inhibitors. Samples were then incubated with rotation at 4 °C for 20 min and centrifuged at 36,000 rpm for 30 min. The supernatant was removed, and the pellet aggressively resuspended in 500 μl of 1 % Triton X-100 buffer (pH 8.0) containing 1 % Triton X-100, 10 mM Tris (pH 8.0), 1 mM EDTA, 1 mM EGTA, 100 μM Na_3_VO_4_, 1 mM NaF, and phosphatase/protease inhibitors (Sigma-Aldrich). Samples were then incubated with rotation at 4 °C for 20 min and further centrifuged at 36,000 rpm for 30 min. The pellet was resuspended in 10 mM Tris containing 25 mg/ml digitonin, 10 mg/ml sodium deoxycholate, 2.5 % NP-40, 1 mM EDTA, 1 mM EGTA, 100 μM Na_3_VO_4_, 1 mM NaF, and phosphatase/protease inhibitors, and the concentration of the resulting PSD enrichments is measured by Lowry assay. Effectiveness of PSD enrichment was assessed by quantification of pre-synaptic proteins synaptophysin and Rab3 in the PSD and membrane fractions from the frontal cortex. Synaptophysin and Rab3 were both abundant in cSM fractions but present at low levels in the PSD (Additional file 1: Figure S1).

### Western blotting

**Fig. 1:**
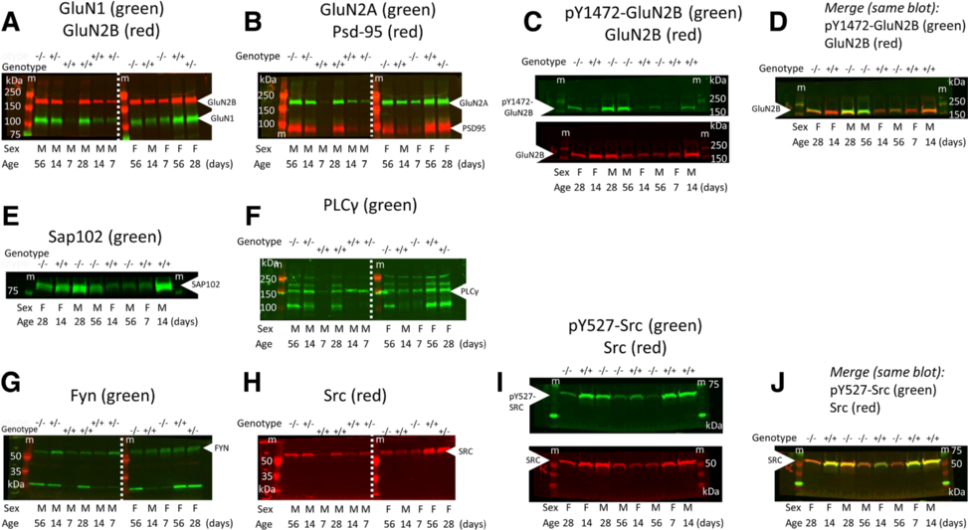
Representative images of Western blots quantified by Odyssey infrared imaging in this study (**a–j**). *M* male, *F* female, *kDa* kilodaltons, *m* marker, −/− DTNBP1 null, +/− DTNBP heterozygous, +/+ wild-type

PSD enrichments (frontal cortex, 2 μg, or hippocampus, 4 μg) or membrane preparations (frontal cortex, 10 μg) were loaded on 7.5 % Tris-glycine polyacrylamide gels (Bio-Rad) and separated by electrophoresis at 200 V. Proteins were then transferred to Immobilon FL PVDF membranes (Millipore, Billerica, MA, USA) at 100 V for 1 h. They were cut at approximately 130 and 75 kDa (as indicated in Fig. 1 and Additional file 1: Figure S2) and after blocking for 1 h in TBS blocking buffer (LI-COR Biosciences, Lincoln, NE, USA), were probed with the following primary antibodies overnight: anti-GluN1 (1:500; sc-1467, Santa Cruz Biotechnology, Dallas, TX, USA); anti-GluN2A (1:1000; sc-1468, Santa Cruz); anti-GluN2B (1:1000; 06-600, Millipore); anti-pY1472-GluN2B (1:500; #4208, Cell Signaling Technology, Danvers, MA, USA); anti-PSD-95 (1:5000; clone K28/43, UC Davis/NIH NeuroMab Facility); anti-SAP102 (1:2000; clone N19/2, UC Davis/NIH NeuroMab Facility); anti-PLCγ1 (1:250; sc-81, Santa Cruz); anti-Fyn (1:500; sc-16, Santa Cruz); anti-cSrc (1:1000; L4A1-2110S, Cell Signaling); anti-pY527-Src (1:500; #2105, Cell Signaling); and anti-dysbindin (1:1000, ab133652, Abcam, Cambridge, UK). Blots were then washed three times for 10 min in Tris-buffered saline containing 0.05 % Tween 20 (TBST) and probed for 1 h with secondary antibodies from LI-COR Biosciences: IRDye 800CW donkey anti-goat (926-32214); IRDye 680LT donkey anti-rabbit (926-68023); IRDye 800CW donkey anti-rabbit (926-32213); IRDye 680RD donkey anti-mouse (926-68072); and IRDye 680LT goat anti-mouse IgG1 specific (926-68050). After three 10-min additional washes in TBST, blots were imaged on the Odyssey infrared imager (LI-COR Biosciences) and band intensities quantified using Odyssey 2.1 software. Images of the entire representative blots for all proteins in this study are provided in Fig. 1 (frontal cortex) and Additional file 1: Figure S2 (hippocampus). Blots were stripped using 1× LI-COR stripping buffer, washed three times in TBS, then reblocked and reprobed. The ranges of linear quantification for the Western blotting assay with Odyssey detection were determined using loading standard curves for each of the proteins measured (Additional file 1: Figure S3, Additional file 1: Figure S4, Additional file 1: Figure S5, Additional file 1: Figure S6). Standard curves were generated (for frontal cortex) using pooled PSD or membrane enrichments from at least four samples of each developmental age, or (for hippocampus) using pooled PSD enrichments from P7 or P56 animals only. Loading amounts within the linear range of the standard curves were used.

### Src/Fyn kinase assay

The activity of Src and Fyn in frontal cortical membrane preparations was determined using a commercially available Src kinase assay kit (Millipore). According to the manufacturers, the substrate is likely to be phosphorylated by both Src and Fyn, given their high homology and the short length of the substrate peptide sequence. Briefly, crude membrane pellets from the frontal cortex were prepared as described above, washed twice with nuclease-free water, and resuspended in nuclease-free water and protein quantified by Lowry assay. Fifteen micrograms of membrane preparation, made up to 10 μl and kept on ice, was added to equal volumes of Src reaction buffer and Src kinase substrate peptide, then warmed briefly to 30 °C. At 15 s intervals, 10 μl of ^32^P-ATP in Mn/ATP cocktail (final ^32^P concentration 1 μCi/μl) was added and mixed and the reaction incubated at 30 °C. After 10 min, reactions were stopped at 15 s interval with 20 μl of 40 % tricholoroacetic acid, before 25 μl was pipetted in duplicates onto P81 phosphocellulose discs. Discs were air dried and washed four times for 5 min with shaking in 0.75 % phosphoric acid, before being transferred into scintillation vials containing 5 ml water for Cerenkov counting in a scintillation counter (Pharmacia). Src/Fyn activity was expressed as picomoles (pmol) (of phosphorylated substrate)/min/μg protein and log transformed before analysis.

### Statistical analysis

All data were either approximately normally distributed (skewness between −1 and 1) or were positively skewed and therefore log transformed prior to analysis (no data were substantially negatively skewed). Sample sizes are indicated in Tables 1 and 2. For quantification of protein abundance for GluN subunits, PSD-95, Src, Fyn, and PLCγ in the frontal cortex, samples were run in duplicate and the geometric mean calculated, whereas for quantification of pY1472-GluN2B, pY527-Src, and SAP102 in the frontal cortex and for all proteins in the hippocampus, samples were run once. For GluN subunits, PSD-95, Src, Fyn, and PLCγ in the frontal cortex, all samples from the cohort were assayed in two Western blotting runs, with half of the samples randomly assigned to each run. For all other assays, samples were run all together in one Western blotting run. In all assays, individual data for each sample was normalized to the average abundance for all samples in the run (i.e., such that, after normalization, the average abundance for samples in the run was 1.0). Data were not further normalized to a loading control, because many commonly used loading controls, such as β-actin, change across development. Instead, where possible, biologically relevant ratios (such as GluN2B:GluN2A, GluN2B:GluN1, and pY1472-GluN2B:total GluN2B), which are inherently internally normalized, were investigated. No outliers were removed. Effects of age group, sex, and genotype were determined using factorial analysis of variance (ANOVA), and relevant specific group differences (after significant ANOVA) were calculated post hoc by least significant difference (LSD) test. Relationships between continuous variables were determined using Pearson’s correlations.

**Table 1:** Summary of sex differences in the frontal cortex

Protein measure	Brain region	N	Number of males and females per age group (P7, 14, 28, 56)	DTNBP1 genotypes included	Sex effect	Males (mean)	Females (mean)	ANOVA *F* statistic (main effect of sex; sex × age interaction)	ANOVA *p* value
PSD enrichments									
GluN2B:GluN2A	FCx	120	15 M, 15 F	−/−, +/−, +/+	M > F	0.97	1.03	Sex *F*(1, 96) = 4.4	*p* < 0.05
								Age *F*(3, 96) = 19.6	*p* < 0.00001
								Interaction *F*(3, 96) = 0.9	N.S.
GluN2B:GluN1	FCx	120	15 M, 15 F	−/−, +/−, +/+	M > F	0.94	1.04	Sex *F*(1, 96) = 8.0	*p =* 0.006
								Age *F*(3, 96) = 4.6	*p* < 0.005
								Interaction *F*(3, 96) = 0.2	N.S.
pY1472-GluN2B: total GluN2B	FCx	64	7–10 M, 6–9 F	−/−, +/+	M > F	0.84	1.14	Sex *F*(1, 48) = 8.0	*p* = 0.007
								Age *F*(3, 48) = 10.8	*p* < 0.00001
								Interaction *F*(,) =	N.S.
Fyn	FCx	120^a^	15 M, 15 F	−/−, +/−, +/+	M > F	0.89	1.01	Sex *F*(1, 96) = 3.8	*p* = 0.055
								Age *F*(3, 96) = 14.1	*p* < 0.00001
								Interaction *F*(3, 96) = 0.3	N.S.
PLCγ	FCx	120	15 M, 15 F	−/−, +/−, +/+	M > F	0.88	1.07	Sex *F*(1, 96) = 8.1	*p =* 0.005
								Age *F*(3, 96) = 1.5	N.S.
								Interaction *F*(3, 96) = 1.2	N.S.
cSM enrichments									
GluN2B:GluN1	FCx	60	9 M, 6 F	−/−, +/−, +/+	M > F	0.93	1.07	Sex *F*(1, 36) = 13.4	*p* < 0.001
								Age *F*(3, 36) = 14.4	*p* < 0.00001
								Interaction *F*(3, 36) = 0.9	N.S.
GluN2A:GluN1	FCx	60	9 M, 6 F	−/−, +/−, +/+	M > F	0.95	1.05	Sex *F*(1, 36) = 8.4	*p* < 0.01
								Age *F*(3, 36) = 58.9	*p* < 0.00001
								Interaction *F*(3, 36) = 1.8	N.S.
PLCγ	FCx	60	9 M, 6 F	−/−, +/−, +/+	M > F	0.88	1.07	Sex *F*(1, 36) = 7.8	*p* < 0.01
								Age *F*(3, 36) = 32.0	*p* < 0.00001
								Interaction *F*(3, 36) = 1.2	N.S.

*N* number of samples, *P* postnatal days, *PSD* postsynaptic density enrichment, *cSM* crude synaptic membrane enrichment, *FCx* frontal cortex, *F* female, *M* male, −/− DTNBP1 null, +/− DTNBP1 heterozygote, +/+ wild-type, *N.S.* not significant

^a^Data log transformed prior to analysis

**Table 2:** Summary of genotype differences in the hippocampus

Protein measure	Brain region	Number	Number of animals of each DTNBP1 genotype per age group^a^ (P7, 14, 28, 56)	Genotype differences	DTNBP1 −/− (null) [mean]	DTNBP1 +/+ (WT) [mean]	ANOVA *F* statistic (main effects of genotype, age and sex; genotype × age interaction, genotype × sex interaction	ANOVA *p* value
PSD enrichments								
GluN2B:GluN1	HIP	80	10 (−/−)10 (−/−)	Genotype × age interaction	P7; 1.04	P7; 1.17	Genotype *F*(1, 64) = 1.9	N.S.
			10 (+/+)		P14; 1.11	P14; 0.95	Age *F*(3, 64) = 7.2	*p* < 0.0005
					P28; 0.99	P28; 0.89	Sex *F*(1, 64) = 0.3	N.S.
					P56; 0.94	P56; 0.88	G × A interaction *F*(3, 64) = 3.9	*p* < 0.05
							G × S interaction *F*(1, 64) = 0.1	N.S.
GluN2A:GluN1	HIP	80	10 (−/−)	DTNBP1(−/−) > WT	1.06	0.94	Genotype *F*(1, 64) = 5.3	*p* < 0.05
			10 (+/+)				Age *F*(3, 64) = 7.9	*p* < 0.0005
							Sex *F*(1, 64) = 1.7	N.S.
							G × A interaction *F*(3, 64) = 0.6	N.S.
							G × S interaction *F*(1, 64) = 0.1	N.S.
Fyn	HIP	80^b^	10 (−/−)	WT > DTNBP1(−/−) females only	F; 0.77	F; 1.24	Genotype *F*(1, 64) = 11.1	*p* < 0.005
			10 (+/+)		M; 0.98	M; 1.03	Age *F*(3, 64) = 25.0	*p* < 0.00001
							Sex *F*(1, 64) = 0.1	N.S.
							G × A interaction *F*(3, 64) = 2.1	N.S.
							G × S interaction *F*(1, 64) = 6.0	*p* < 0.05
pY527-Src	HIP	80	10 (−/−)	WT > DTNBP1(−/−)	0.92	1.08	Genotype *F*(1, 64) = 5.7	*p* < 0.05
			10 (+/+)				Age *F*(3, 64) = 21.0	*p* < 0.00001
							Sex *F*(1, 64) = 0.1	N.S.
							G × A interaction *F*(3, 64) = 1.0	N.S.
							G × S interaction *F*(1, 64) = 3.6	(*p* = 0.06)
PLCγ	HIP	80	10 (−/−)	WT > DTNBP1(−/−)	0.97	1.02	Genotype *F*(1, 64) = 5.1	*p* < 0.05
			10 (+/+)				Age *F*(3, 64) = 2.5	N.S.
							Sex *F*(1, 64) = 1.7	N.S.
							G × A interaction *F*(3, 64) = 2.3	N.S.
							G × S interaction *F*(1, 64) = 0.1	N.S.

*N* number of samples, *P* postnatal days, *PSD* postsynaptic density enrichments, *HIP* hippocampus, *WT* wild-type, *G* genotype, *A* age, *S* sex, *N.S.* not significant

^a^Includes both males and females in each age/genotype group

^b^Data log transformed prior to analysis

## Results

### GluN2B-GluN2A switch in the PSD of the frontal cortex and hippocampus

**Fig. 2:**
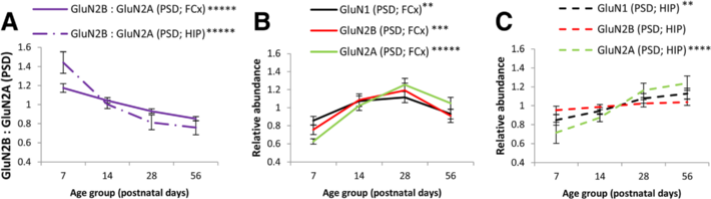
The GluN2B-GluN2A switch in PSD enrichments from the frontal cortex and hippocampus. **a** Proportion of GluN2B relative to GluN2A (GluN2B:GluN2A) in PSD enrichments from the frontal cortex and hippocampus. **b** Levels of GluN1, GluN2A, and GluN2B in PSD fractions from the frontal cortex. **c** Levels of GluN1, GluN2A, and GluN2B in PSD fractions from the hippocampus. **p ≤ 0.005; ***p < 0.0005; ****p ≤ 0.0001; *****p < 0.00001. *PSD* postsynaptic density, *FCx* frontal cortex, *HIP* hippocampus. *Error bars* represent standard error of the mean (SEM)

The GluN2B-GluN2A switch was observed in the frontal cortex and hippocampus in the form of progressive decreases in the ratio of GluN2B:GluN2A in PSD enrichments (frontal cortex *F*(3, 96) = 19.6, *p* < 1 × 10^−5^; hippocampus *F*(3, 64) = 17.5, *p* < 1 × 10^−5^; Fig. 2a). This GluN2B-GluN2A switch was more gradual and protracted in the frontal cortex than in the hippocampus. It occurred in the context of developmental increases in the abundance of all GluN subunits in the frontal cortex (GluN1 *F*(3, 96) = 4.4, *p* = 0.005; GluN2A *F*(3, 96) = 19.4, *p* < 1 × 10^−5^; GluN2B *F*(3, 96) = 7.3, *p* < 0.0005; Fig. 2b) and GluN1 and GluN2A in the hippocampus (GluN1 *F*(3, 64) = 5.8, *p* < 0.005; GluN2A *F*(3, 64) = 8.8, *p* < 0.0001; GluN2B *F*(3, 64) = 1.1, *p* = 0.40; Fig. 2c).

Other proteins in this study also changed across postnatal life. Main effects of age for other proteins in the PSD in this study are illustrated in Additional file 1: Figure S7, including Src and PLCγ, the only proteins in this study whose developmental expression patterns differed substantially between PSD enrichments and cSM fractions.

### Sex differences in the GluN2B-GluN2A switch and associated NMDA receptor signaling proteins at the frontal cortical synapse

**Fig. 3:**
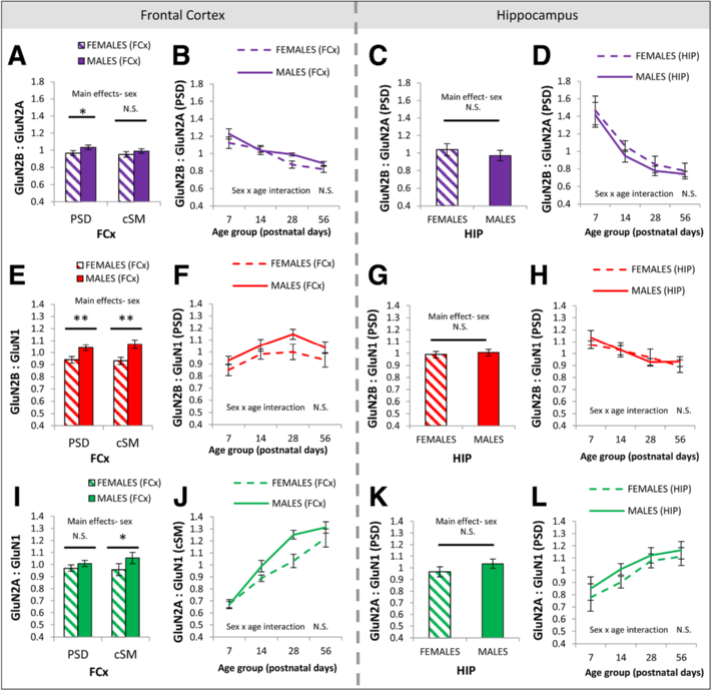
Sex differences in NMDA receptor signaling proteins in PSD and cSM enrichments of the frontal cortex and hippocampus. **a**, **e**, **i** Main effects of sex on GluN2B:GluN2A, GluN2B:GluN1, and GluN2A:GluN1 ratios in PSD and cSM enrichments of the frontal cortex. **b**, **f**, **j** Developmental trajectories of GluN2B:GluN2A, GluN2B:GluN1, and GluN2A:GluN1 ratios in females and males in the PSD of the frontal cortex. **c**, **g**, **k** Main effects of sex on GluN2B:GluN2A, GluN2B:GluN1, and GluN2A:GluN1 ratios in PSD enrichments of the hippocampus. **d**, **h**, **l** Developmental trajectories of GluN2B:GluN2A, GluN2B:GluN1, and GluN2A:GluN1 ratios in females and males in the PSD of the hippocampus. Images of representative blots for all hippocampal proteins are provided in Additional file 1: Figure S3. For bar graphs in panels **a**, **c**, **e**, **g**, **i**, and **k**, data are collapsed across age groups. Refer to Table 1 for sample sizes. *PSD* postsynaptic density, *cSM* crude synaptic membrane, *FCx* frontal cortex, *N.S.* not significant. *p < 0.05; **p ≤ 0.006. *Error bars* represent SEM

The balance of GluN2B and GluN2A subunits differed between males and females across postnatal development in the frontal cortex (Fig. 3a, b, e, f, i, j) but not the hippocampus (Fig. 3c, d, g, h, k, l). A main effect of sex was seen in the ratio of GluN2B:GluN2A in PSD enrichments of the frontal cortex (*F*(1, 96) = 4.4, *p* < 0.05; Fig. 3a), with an overall increase in the GluN2B:GluN2A ratio in males. This difference was evident broadly across development, with no interaction between sex and age for GluN2B:GluN2A in the PSD (*F*(3, 96) = 0.9, *p* = 0.43; Fig. 3b). To determine whether increases in GluN2B:GluN2A in males arose due to differences in postsynaptic density abundance of GluN2B or GluN2A specifically, we quantified the GluN2B:GluN1 and GluN2A:GluN1 ratios. These ratios represent the abundance of each GluN2 subunit relative to the total abundance of NMDA receptors (which all contain the obligatory GluN1 subunit). The sex difference in the GluN2B:GluN2A ratio in PSD enrichments from the frontal cortex appeared to be driven by differences in levels of GluN2B, with a sex difference also observed in the GluN2B:GluN1 ratio (*F*(1, 96) = 8.0, *p* = 0.0057; Fig. 3e) but not the GluN2A:GluN1 ratio (*F*(1, 96) = 2.4, *p* = 0.13; Fig. 3i). There were no interactions between sex and age for GluN2B:GluN1 (*F*(3, 96) = 0.2, *p* = 0.88; Fig. 3f) or GluN2A:GluN1 (*F*(3, 96) = 0.4, *p* = 0.73; Fig. 3j) in the PSD. The observed sex differences were present in the wild-type, heterozygous DTNBP1 mutants and homozygous DTNBP1 mutant mice, with no interactions of sex and genotype observed for GluN2B:GluN2A (*F*(2, 96) = 0.1, *p* = 0.88) or GluN2B:GluN1 (*F*(2, 96) = 0.03, *p* = 0.97). Sex differences in abundance of GluN2B and GluN2A subunits were also detectable cSM enrichments. As in PSD enrichments, the GluN2B:GluN1 ratio was greater in males than females in cSM enrichments from the frontal cortex (*F*(1, 36) = 13.44, *p* < 0.001; Fig. 3e), but this was balanced by an increase in the GluN2A:GluN1 ratio in membranes in males compared to females (*F*(1, 36) = 8.4, *p* < 0.01; Fig. 3i). As a result, no sex differences were seen in the GluN2B:GluN2A ratio in frontal cortical membranes (*F*(1, 36) = 1.8, *p* = 0.19; Fig. 3a). In the hippocampus, there were no sex differences in the ratios of GluN2B:GluN2A (main effect *F*(1, 64) = 0.9, *p* = 0.35; Fig. 3c), GluN2B:GluN1 (main effect *F*(1, 64) = 0.3, *p* = 0.61; Fig. 3g), or GluN2A:GluN1 (main effect *F*(1, 64) = 1.7, *p* = 0.19; Fig. 3k).

**Fig. 4:**
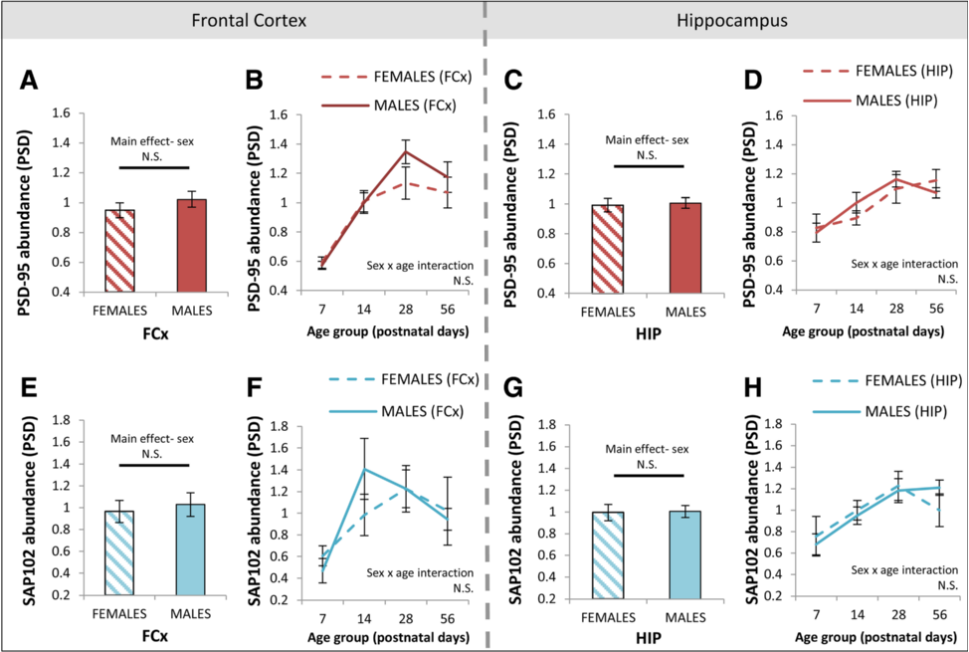
Absence of sex differences in scaffolding proteins in PSD enrichments of the frontal cortex. **a**, **e** Main effects of sex on the abundance of PSD-95 and SAP102 in PSD enrichments of the frontal cortex. **b**, **f** Developmental trajectories of PSD-95 and SAP102 in females and males in the PSD of the frontal cortex. **c**, **g** Main effects of sex on the abundance of PSD-95 and SAP102 in PSD enrichments of the hippocampus. **d**, **h** Developmental trajectories of PSD-95 and SAP102 in females and males in the PSD of the hippocampus. *PSD* postsynaptic density, *cSM* crude synaptic membrane, *FCx* frontal cortex, *N.S.* not significant. *Error bars* represent SEM

Next, we investigated three possible mechanisms which could contribute to the observed sex differences in GluN2B at the synapse in the frontal cortex. Firstly, we quantified PSD scaffolding proteins PSD-95 and SAP102, which regulate NMDA receptor synaptic targeting and clearance [94–96] and may preferentially bind GluN2B-containing and GluN2A-containing NMDA receptors, respectively [8]. There were no main effects of sex on the abundance of PSD-95 (*F*(1, 96) = 1.48, *p* = 0.23; Fig. 4a) or SAP102 (*F*(1, 48) = 0.22, *p* = 0.64; Fig. 4e) in PSD enrichments from the frontal cortex or interactions of sex × age for these scaffolding proteins, suggesting that differences in the postsynaptic architecture were not driving sex differences in GluN2 subunit balance across postnatal life. There were no sex differences in PSD-95 (*F*(1, 64) = 0.1, *p* = 0.79; Fig. 4c) or SAP102 (*F*(1, 64) = 0.01, *p* = 0.91; Fig. 4g) in the hippocampal PSD enrichments.

**Fig. 5:**
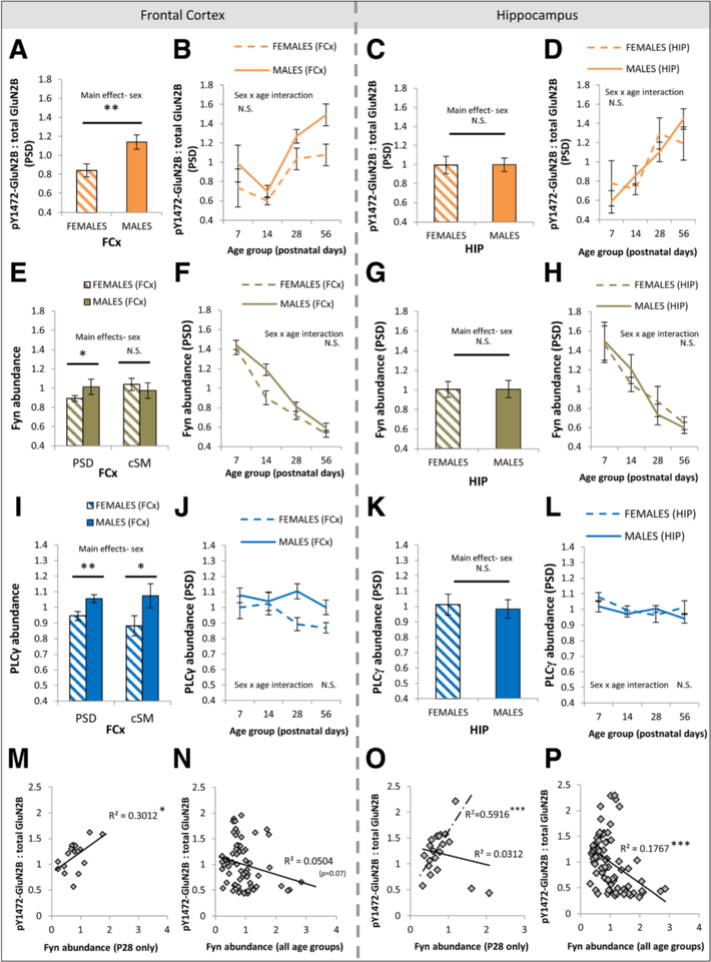
Sex differences in pY1472-GluN2B phosphorylation, Fyn, and PLCγ in PSD enrichments of the frontal cortex. **a**, **e**, **i** Main effects of sex on the pY1472-GluN2B:total GluN2B ratio, abundance of Fyn, and abundance of PLCγ in PSD and cSM enrichments of the frontal cortex. **b**, **f**, **j** Developmental trajectories of pY1472-GluN2B:total GluN2B ratio, Fyn, and PLCγ in females and males in the PSD of the frontal cortex. **c**, **g**, **k** Main effects of sex the pY1472-GluN2B:total GluN2B ratio, abundance of Fyn, and abundance of PLCγ in PSD enrichments of the hippocampus. **d**, **h**, **l** Developmental trajectories of the pY1472-GluN2B:total GluN2B ratio, Fyn, and PLCγ in females and males in the PSD of the hippocampus. **m** Negative correlation of levels of Fyn with pY1472-GluN2B:total GluN2B ratio in PSD enrichments of the frontal cortex across all age groups. **n** Positive correlation of levels of Fyn with pY1472-GluN2B:total GluN2B ratio in PSD enrichments of the frontal cortex at P28 only. **o** Negative correlation of levels of Fyn with pY1472-GluN2B:total GluN2B ratio in PSD enrichments of the hippocampus across all age groups. **p** Correlation of levels of Fyn with pY1472-GluN2B:total GluN2B ratio in PSD enrichments of the hippocampus at P28 only (*dotted line* indicates positive correlation when two outlying data points are excluded). *PSD* postsynaptic density, *cSM* crude synaptic membrane, *FCx* frontal cortex, *N.S*. not significant. *p ≤ 0.055; **p ≤ 0.007. *Error bars* represent SEM

Secondly, phosphorylation of GluN2B at tyrosine 1472 is a well-characterized mechanism which facilitates the membrane localization of GluN2B subunit-containing NMDA receptor complexes via the inhibition of endocytosis [94, 97, 98]. We quantified pY1472-GluN2B to determine whether differences between females and males in Y1472-GluN2B phosphorylation in the frontal cortex may underlie sex differences in GluN2 subunit balance. There was a main effect of sex on the ratio of pY1472-GluN2B to the total GluN2B in PSD enrichments (*F*(1, 48) = 8.0, *p =* 0.0068; Fig. 5a). Males had a greater proportion of GluN2B phosphorylated at Y1472 than females. Qualitatively, this sex difference was greatest in adults (Fig. 5b). No sex difference in Y1472-GluN2B phosphorylation was seen in the PSD of the hippocampus (*F*(1, 64) = 0.01, *p* = 0.98; Fig. 5c).

Increased pY1472-GluN2B in the frontal cortex may result from increased abundance or activity of tyrosine kinases Fyn and Src, which phosphorylate GluN2 subunits [99, 100]. Evidence suggests that Fyn specifically phosphorylates GluN2B at Y1472 [100]. There was a subtle main effect of sex on Fyn abundance in the PSD of the frontal cortex (*F*(1, 96) = 3.8, *p* = 0.055; Fig. 5e). As predicted by increased pY1472-GluN2B, Fyn was increased in males relative to females. There were no sex differences in Fyn abundance in cSM fractions from the frontal cortex (*F*(1, 36) = 0.38, *p* = 0.54; Fig. 5e). There were no sex differences in the abundance of Src (*F*(1, 96) = 0.0004, *p* = 0.98) or in the relative proportion of “inactive” Src phosphorylated at tyrosine 527 (pY527-Src:total Src) (*F*(1, 48) = 10.26, *p =* 0.27) in PSD enrichments from the frontal cortex. Quantification of Src and Fyn activities, using a Src family kinase activity assay, did not reveal an effect of sex on the overall activity of Src and Fyn together in cSM fractions from the frontal cortex (*F*(1, 84) = 0.50, *p* = 0.48). There were no sex differences in Fyn (*F*(1, 64) = 0.1, *p* = 0.73; Fig. 5g) or Src (*F*(1, 64) = 0.01, *p* = 0.97) in the PSD of the hippocampus. A relationship between the levels of Fyn and pY1472-GluN2B at the synapse was supported by a positive correlation of Fyn with the ratio of pY1472-GluN2B to the total GluN2B at P28 in the frontal cortex (*p* < 0.05; Fig. 5m), during the developmental period when the observed sex differences are becoming more apparent. Although there was no such positive correlation in the hippocampus when all P28 samples were analyzed (Fig. 5o, solid line), there was a strong positive correlation if two outliers were omitted from the analysis (Fig. 5o, dotted line). In contrast, negative correlations were seen between Fyn and pY1472-GluN2B:total GluN2B across postnatal life as a whole in the frontal cortex and (particularly) in the hippocampus (Fig. 5n, p), suggesting that Fyn levels were not driving overall developmental changes in pY1472-GluN2 phosphorylation.

Finally, we investigated phospholipase C (PLC) at the synapse. PLC is critical to the GluN2B-GluN2A switch, driving activity-dependent, mGluR5-mediated increases in GluN2A [21]. In an apparent contradiction, PLCγ has also been reported to facilitate GluN2B phosphorylation [101] and bind to phosphorylated GluN2B [102]. As a result, differences in its abundance or function may contribute to sex differences in the GluN2B-GluN2A switch. PLCγ was different between males and females in PSD enrichments from the frontal cortex (*F*(1, 96) = 8.1, *p* = 0.005; Fig. 5i), being increased in males compared to females. This was mirrored in the cSM fraction of the frontal cortex (*F*(1, 36) = 7.8, *p* < 0.01; Fig. 5i). Qualitatively, the greatest sex difference in levels of PLCγ occurred in the PSD in pre-adolescent animals (P28; Fig. 5j). There were no sex differences in PLCγ (*F*(1, 64) = 1.7, *p* = 0.19; Fig. 5k) in the PSD of the hippocampus. Significant sex differences in the frontal cortex are summarized in Table 1.

### Effects of DTNBP1 null mutation on the GluN2B-GluN2A switch at the hippocampal synapse

**Fig. 6:**
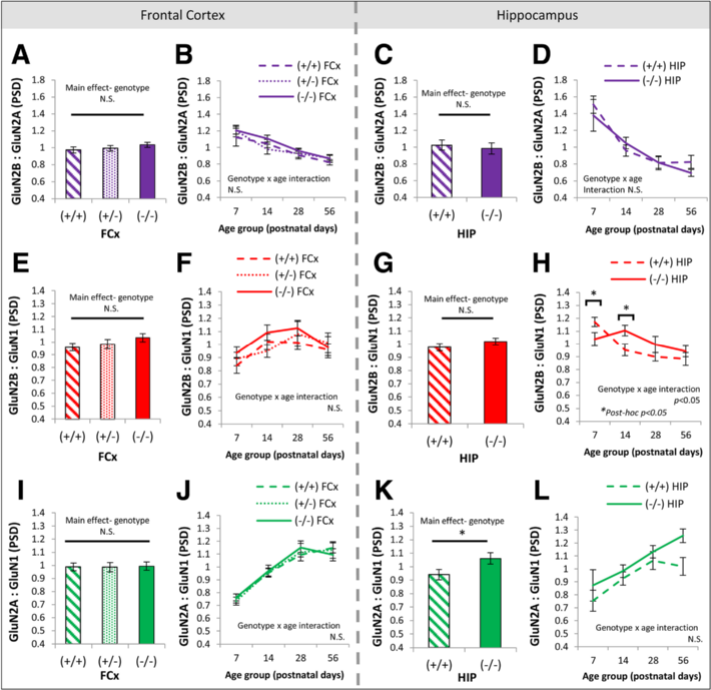
Effects of DTNBP1 genotype on the GluN2B-GluN2A switch in the frontal cortex and hippocampus. **a**, **e**, **i** Main effects of DTNBP1 genotype [wild-type (+/+), heterozygous DTNBP1 mutant (+/−), and homozygous DTNBP1 null mutant (−/−)] on GluN2B:GluN2A, GluN2B:GluN1, and GluN2A:GluN1 ratios in PSD enrichments of the frontal cortex. **b**, **f**, **j** Developmental trajectories of GluN2B:GluN2A, GluN2B:GluN1, and GluN2A:GluN1 ratios in wild-type (+/+), heterozygous DTNBP1 mutant (+/−), and homozygous DTNBP1 null mutant (−/−) mice in the PSD of the frontal cortex. **c**, **g**, **k** Main effects of DTNBP1 genotype [wild-type (+/+) and homozygous DTNBP1 null mutant (−/−)] on GluN2B:GluN2A, GluN2B:GluN1, and GluN2A:GluN1 ratios in PSD enrichments of the hippocampus. **d**, **h**, **l** Developmental trajectories of GluN2B:GluN2A, GluN2B:GluN1, and GluN2A:GluN1 ratios in wild-type (+/+) and homozygous DTNBP1 null mutant (−/−) mice in the PSD of the hippocampus. Images of representative blots for all hippocampal proteins are provided in Additional file 1: Figure S3. For bar graphs in panels **a**, **c**, **e**, **g**, **i**, and **k**, data are collapsed across age groups. Refer to Table 2 for sample sizes. *PSD* postsynaptic density, *cSM* crude synaptic membrane, *FCx* frontal cortex, *N.S.* not significant. *p < 0.05. *Error bars* represent SEM

The GluN2B-GluN2A switch was also impacted by DTNBP1 genotype, with differences between DTNBP(−/−) and WT mice evident in the hippocampus (Fig. 6c, d, g, h, k, l) but not in the frontal cortex (Fig. 6a, b, e, f, i, j). There was not a main effect of genotype on the GluN2B:GluN2A ratio (*F*(1, 64) = 0.3, *p* = 0.56; Fig. 6c) or an interaction between genotype and age for the GluN2B:GluN2A ratio in the hippocampal PSD (*F*(3, 64) = 0.6, *p* = 0.64; Fig. 6d). However, at the level of individual GluN2 subunits, there was a disruption of the normal developmental trajectory of GluN2B in DTNBP1(−/−) mice, seen in an interaction between genotype and age for the ratio of GluN2B:GluN1 (*F*(3, 64) = 3.85, *p* < 0.05; Fig. 6h). WT mice showed developmental decreases in the GluN2B:GluN1 from P7 to P14 (*p* < 0.005) but DTNBP1(−/−) mice did not (*p* = 0.28; Fig. 6h). As a result, the GluN2B:GluN1 ratio was increased in WT mice relative to DTNBP1(−/−) mice at P7 (*p* < 0.05), but the reverse was true at P14 (*p* < 0.05). These developmental differences did not result in an overall difference in GluN2B:GluN1 ratios between DTNBP1(−/−) and WT mice across development as a whole (main effect genotype *F*(1, 64) = 1.9, *p* = 0.17; Fig. 6g). There was an overall increase in the proportion of GluN2A relative to GluN1 in DTNBP1(−/−) mice in hippocampal PSD enrichments across development (main effect genotype *F*(1, 64) = 5.3, *p* < 0.05; Fig. 6k), which was qualitatively strongest in adulthood (Fig. 6l), by which time GluN2B levels in DTNBP1(−/−) mice were approaching WT levels (Fig. 6h). In the frontal cortex, there were no main effects of genotype on the GluN2B:GluN2A ratio (*F*(2, 96) = 1.3, *p* = 0.27; Fig. 6a), GluN2B:GluN1 ratio (*F*(2, 96) = 1.6, *p* = 0.20; Fig. 6e), or the GluN2A:GluN1 ratio (*F*(2, 96) = 0.05, *p* = 0.95; Fig. 6i) in PSD enrichments.

**Fig. 7:**
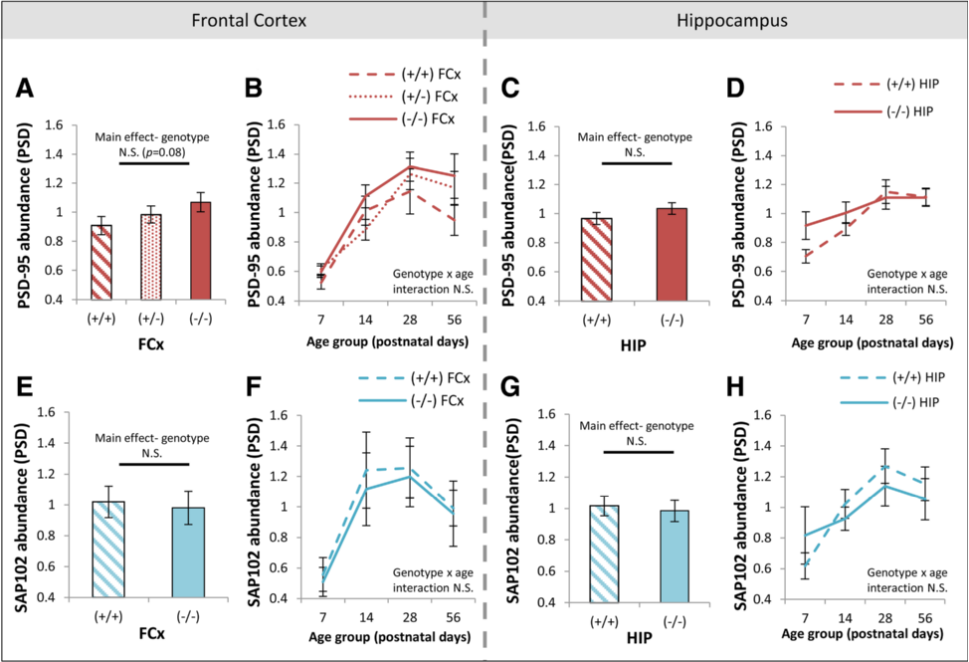
Absence of DTNBP1 genotype differences in scaffolding proteins in PSD enrichments of the frontal cortex and hippocampus. **a**, **e** Main effects of DTNBP1 genotype on abundance of PSD-95 and SAP102 in PSD enrichments of the frontal cortex. **b** Developmental trajectories of PSD-95 in wild-type (+/+), heterozygous DTNBP1 mutant (+/−), and homozygous DTNBP1 null mutant (−/−) mice in the PSD of the frontal cortex. **f** Developmental trajectories of SAP102 in wild-type (+/+) and homozygous DTNBP1 null mutant (−/−) mice in the PSD of the frontal cortex. **c**, **g** Main effects of DTNBP1 genotype [wild-type (+/+) and homozygous DTNBP1 null mutant (−/−)] on abundance of PSD-95 and SAP102 in PSD enrichments of the hippocampus. **d**, **h** Developmental trajectories of PSD-95 and SAP102 in wild-type (+/+) and homozygous DTNBP1 null mutant (−/−) mice in the PSD of the hippocampus. *PSD* postsynaptic density, *cSM* crude synaptic membrane, *FCx* frontal cortex, *N.S.* not significant. *Error bars* represent SEM

**Fig. 8:**
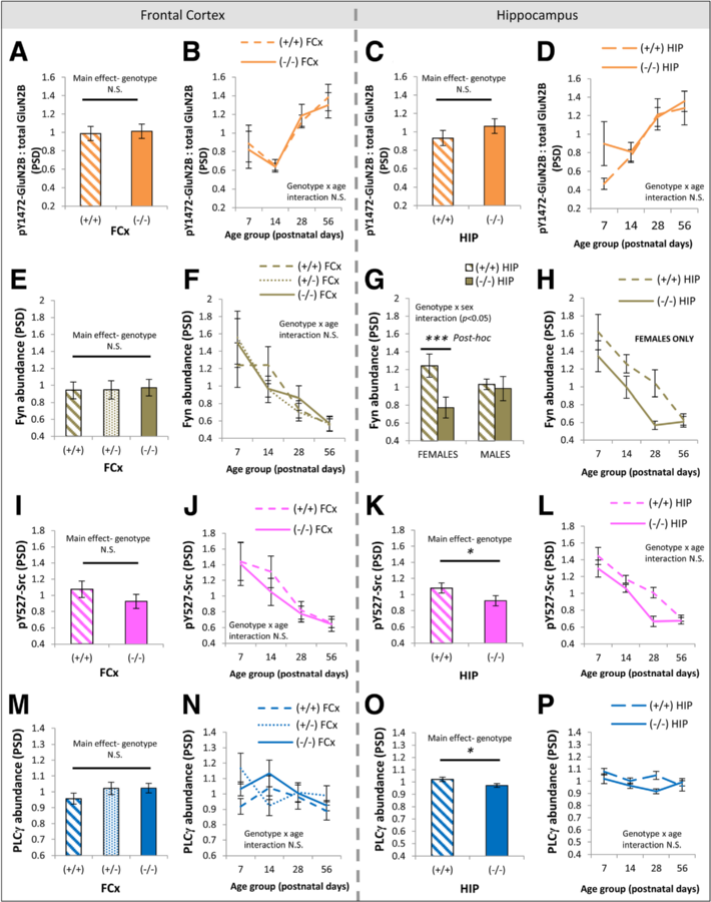
DTNBP1 genotype differences in pY1472-GluN2B phosphorylation, Fyn, and PLCγ in PSD enrichments of the frontal cortex and hippocampus. **a**, **e**, **i**, **m** Main effects of DTNBP1 genotype on the pY1472-GluN2B:total GluN2B ratio, abundance of Fyn, pY527-Src phosphorylation, and abundance of PLCγ in PSD enrichments of the frontal cortex. **b**, **f**, **j**, **n** Developmental trajectories of pY1472-GluN2B:total GluN2B ratio, abundance of Fyn, pY527-Src phosphorylation, and abundance of PLCγ in mice of different DTNBP1 genotypes in the PSD of the frontal cortex. **c**, **g**, **k**, **o** Main effects of DTNBP1 genotype on the pY1472-GluN2B:total GluN2B ratio, abundance of Fyn, pY527-Src phosphorylation, and abundance of PLCγ in PSD enrichments of the hippocampus. **d**, **h**, **l**, **p** Developmental trajectories of pY1472-GluN2B:total GluN2B ratio, abundance of Fyn, pY527-Src phosphorylation, and abundance of PLCγ in mice of different DTNBP1 genotypes in the PSD of the hippocampus. *p < 0.05; ***p ≤ 0.0005. *N.S.* not significant. *Error bars* represent SEM

Accompanying the developmental disruption of hippocampal GluN2B in DTNBP1 mutant mice, there were no main effects of DTNBP1 genotype on scaffolding proteins PSD-95 (*F*(1, 64) = 1.8, *p* = 0.17; Fig. 7c) or SAP102 (*F*(1, 64) = 0.1, *p* = 0.71; Fig. 7g) or the extent of pY1472-GluN2B phosphorylation (*F*(1, 64) = 1.67, *p* = 0.20; Fig. 8c) in hippocampal PSD enrichments. However, there was a main effect of DTNBP1 genotype on the abundance of Fyn (*F*(1, 64) = 11.1, *p* < 0.005), where Fyn levels were decreased in homozygous DTNBP1 mutant mice compared to wild-types. This genotype effect was modified by sex (genotype × sex interaction *F*(1, 64) = 6.0, *p* < 0.05). A strong genotype effect was seen in female animals (*p* < 0.0005; Fig. 8g) up until adulthood (Fig. 8h) but not in males (*p* = 0.9). Two additional proteins in PSD enrichments from the hippocampus were impacted by DTNBP1 genotype. Phosphorylation of Src at pY527 (which leads to its folding into an inactive state [103]) was decreased in DTNBP1(−/−) mice (*F*(1, 64) = 5.7, *p* < 0.05; Fig. 8k), predominantly up until adulthood (Fig. 8l). Similarly, PLCγ levels were decreased in DTNBP1(−/−) mice (*F*(1, 64) = 5.1, *p* < 0.05; Fig. 8o), with the genotype difference most apparent qualitatively at P28 (Fig. 8p). To determine how hippocampal levels of dysbindin 1 (DTNBP1) protein vary across postnatal life, and whether they are highest early in life as reported in whole brain homogenates [66], we investigated dysbindin in our hippocampal PSD enrichments. We were not able to reliably detect dysbindin 1A or dysbindin 1C in hippocampal PSD enrichments using the Abcam ab133652 antibody (data not shown). In the frontal cortex, there were no main effects of genotype on levels of PSD-95 (*F*(2, 96) = 2.6, *p* = 0.08; Fig. 7a), SAP102 (*F*(1, 48) = 0.02, *p* = 0.89; Fig. 7e), pY1472-GluN2B phosphorylation (*F*(1, 48) = 0.02, *p* = 0.88; Fig. 8a), Fyn (*F*(2, 96) = 0.08, *p* = 0.92; Fig. 8e), pY527-Src phosphorylation (*F*(1, 48) = 0.25, *p* = 0.62; Fig. 8i), or PLCγ (*F*(2, 96) = 1.3, *p* = 0.27; Fig. 8m) in the PSD. A summary of genotype differences in the PSD of the hippocampus is contained in Table 2.

## Discussion

In this study, we provide molecular evidence that the developmental shift in the balance of GluN2B and GluN2A in the cortex differs in females and males and in the hippocampus, can be disrupted by DTNBP1 null mutation. In males compared to females, the balance of GluN2 subunits was shifted toward a greater abundance of GluN2B-containing NMDA receptors at the cortical synapse, accompanied by underlying increases in Y1472-GluN2B phosphorylation, Fyn tyrosine kinase abundance, and PLCγ abundance. In the hippocampus, the developmental trajectory of GluN2B maturation was disrupted in DTNBP1(−/−) mice compared to WT mice, alongside increases in the relative abundance of GluN2A. These changes occurred in the context of genotype effects on Y527-Src phosphorylation, Fyn abundance in females only, and PLCγ abundance. Overall, these findings suggest that sex and DTNBP1 genotype can influence GluN2 subunit balance, in a process possibly involving Fyn and PLCγ. They also support the hypothesis that differences in GluN2 subunit balance, arising developmentally during the GluN2B-GluN2A switch, may underlie some sex- or genotype-related individual differences in risk for neurodevelopmental disorders.

It is interesting to note that the molecules that showed sex differences at the cortical synapse may be related functionally and mechanistically. Males had more abundant representation of Fyn and PLCγ, and higher pY1472-GluN2B:total GluN2B, GluN2B:GluN2A, and GluN2B:GluN1 ratios, than females in PSD enrichments from the frontal cortex. In the pre-adolescent period, the increased synaptic abundance in males of the tyrosine kinase Fyn, which phosphorylates GluN2B at Y1472 [100], may lead to male-specific increases in Y1472-GluN2B phosphorylation. This in turn may lead to increased stability of GluN2B-containing NMDA receptors at the synapse in males [94, 97, 98], reflected in increased GluN2B:GluN2A and GluN2B:GluN1 ratios in the PSD. Consistent with this scenario, we observed that levels of Fyn in the PSD were positively correlated with pY1472-GluN2B:total GluN2B ratio within the P28 age group in the frontal cortex. However, at P28 when qualitative sex differences in Fyn were greatest, the sex differences in pY1472-GluN2B phosphorylation were most subtle. Furthermore, Fyn was negatively correlated with pY1472-GluN2B phosphorylation across the lifespan as a whole, decreasing with postnatal age while pY1472-GluN2B phosphorylation increased. This suggests that, while Fyn may fine-tune levels of GluN2B phosphorylation in pre-adolescence, other mechanisms may also contribute to sex differences and drive the developmental increase pY1472-GluN2B phosphorylation across postnatal life as a whole. Alternatively, increased pY1472-GluN2B phosphorylation in males may also arise from increased abundance or activity of PLCγ, which can facilitate GluN2B phosphorylation [101], was increased in males at the ages when pY1472-GluN2B was also different in males, and was positively correlated with pY1472-GluN2B levels at P28. Sex differences in PLCγ may also indicate a compensatory change, aimed at driving mGluR5-mediated increases in GluN2A to “normalize” the increased relative abundance of GluN2B. The emergence of sex differences in the GluN2B:GluN2A ratio, pY1472-GluN2B:total GluN2B ratio, and PLCγ abundance in the PSD appeared to coincide with the juvenile (P14-P28) period, prior to the pubertal surge in sex hormones during adolescence. This suggests that the divergence of males and females may be due to organizational effects of sex hormones in males during the early life surge in testosterone or due to sex differences in autosomal gene/protein expression [10, 104]. The emergence of sex differences during discrete developmental epochs reinforces the value of using developmental trajectories, rather than isolated developmental endpoints, to assess molecular parameters of relevance to brain function and behavior.

Further studies are required to understand the consequences of sex differences in GluN2B-GluN2A balance in the PSD of the frontal cortex. Typically, GluN2B-containing NMDA receptors have been viewed as most critical to healthy brain function early in postnatal development (coincident with their high relative abundance), during which time they are involved in synapse formation, stabilization, and plasticity [105, 106]. Later in life, GluN2B-containing NMDA receptors have been considered key contributors to extra-synaptic NMDA receptor signaling, responsible for the damaging excitotoxicity which occurs in response to brain injury [107] (for review, see [108, 109]). In this study, however, relative GluN2B abundance and phosphorylation were increased in males during adolescence and/or adulthood in PSD enrichments (i.e., predominantly within the synapse), suggesting that any effects of these sex differences would be mediated via synaptic actions of GluN2B-containing NMDA receptor complexes later in life. It is possible that GluN2B-related sex differences may impact a substantial proportion of NMDA receptors in the adult cortex if tri-heteromeric GluN1/GluN2A/GluN2B-containing NMDA receptors are more common than previously thought, as has been indicated in the hippocampus [110]. Indeed, GluN2B-containing NMDA receptors have recently been shown to play a critical role in layer 5 pyramidal neurons of the prefrontal cortex in adulthood, a role which is acquired during adolescence [111]. Throughout the lifespan, pY1472 phosphorylation may also be important, as it is modulated by NMDA receptor activity [112], mediates some of the detrimental effects of neonatal hypoxic brain injury [113], and may play a role in pathological processes such as alcohol dependency [114, 115]. Future work exploring whether observations in this study are driven by sex differences in specific cell types, such as pyramidal neurons or interneurons, may also shed light on the possible functional consequences of such differences and their relevance to ASD, schizophrenia, or other neurodevelopmental disorders.

Future work may also determine whether sex differences in GluN2B-GluN2A balance, as observed here in rodents, also exist in the human cortex, and if such sex differences relate to other sex differences in the brain structure, neural activity, cognitive function, and risk for psychiatric illness. The brains of males and females have been found to have differences in molecular [104, 116, 117], structural [118–128], and functional [129–133] components. Females and males are also differentially susceptible to neurodevelopmental disorders such as ASD [55, 56] and schizophrenia [48, 49, 59], as well as other psychiatric illnesses such as major depressive disorder [134, 135]. In experimental settings, male and female rodents are differentially sensitive to disturbance of brain circuits and behavior following postnatal ketamine, cocaine, morphine, and nicotine exposure [136–139]. Interestingly, males have more severe behavioral deficits than females in a rodent model of NMDA receptor deficiency [140] and after early postnatal NMDA receptor blockade [141]. If the sex differences observed here extend to humans, it is plausible that pathophysiologic mechanisms in neurodevelopmental disorders which converge on GluN2A- or GluN2B-mediated NMDA receptor signaling may differentially impact males and females.

In this study, we identified effects of DTNBP1 mutation on the GluN2B-GluN2A shift, particularly on the developmental trajectory of GluN2B and the overall synaptic abundance of GluN2A in the hippocampus. Dysbindin 1 (protein product of the DTNBP1 gene) is a component of the BLOC-1 complex and has diverse functions in the brain including supporting dendritic spine formation and facilitating glutamate release (for review, see [142]). In the whole cortex, dysbindin 1A expression has been reported to decrease across postnatal development, particularly up to P28 [66]. In our study, the greatest disruption to GluN2B at the PSD occurred up to P28, after which time, the GluN2B:GluN1 ratio in DTNBP1(−/−) mice approached WT levels. We also observed increased overall GluN2A in DTNBP1(−/−) mice across postnatal life in the hippocampal PSD. However, we did not observe a main effect of genotype effects in the GluN2B:GluN2A ratio, potentially as a result of concurrent differences at some developmental ages in both GluN2B and GluN2A. Our findings with GluN2A mirror previous work in DTNBP1(−/−) mice [84], in which GluN2A surface expression was increased in cultured hippocampal neurons from DTNBP1(−/−) mice (harvested at E18, cultured 16 days), hippocampal EPSCs were increased with faster decay time in pre-adolescent DTNBP1(−/−) mice (P26–31), and hippocampal long-term potentiation (LTP) was increased in adult DTNBP1(−/−) mice (P56) [84]. However, a contradictory decrease in LTP in adult DTNBP1(−/−) mice at P35–50 and P90–120 has also been reported [87]. Decreased amplitude of NMDA-evoked currents in cortical neurons at P45–60, accompanied by decreased GRIN1 gene expression and impaired working memory, has also been described [83]. Although some of this previous work identified frontal cortex-related deficits, our work suggests that the hippocampus may be more sensitive than the frontal cortex to the effects of DTNBP1 mutation on the GluN2B-GluN2A switch, as assessed using our techniques. Our findings are consistent with work investigating another schizophrenia risk factor, neuregulin 1. Neuregulin 1 (Nrg1) is a candidate schizophrenia risk gene [143] which has been found to increase GluN2B phosphorylation via PLCγ-dependent mechanism in cortical neurons [101]. This process involves ErbB4-TrkB interaction, which is decreased in the brain in individuals with schizophrenia [101]. In Nrg1 transmembrane heterozygous knockout mice, pY1472-GluN2B is reduced, in parallel with increased baseline and decreased evoked power of gamma frequency neural oscillations, reproducible features of schizophrenia [144]. Future work may determine whether GluN2B phosphorylation, and/or other developmental events related to the GluN2B-GluN2A switch, represent points of convergence for putative risk genes for neurodevelopmental disorders. Overall, our findings highlight the potential for genetic variants in DTNBP1 and other developmentally regulated genes to influence maturational programs, such as the GluN2B-GluN2A switch, which are relevant to neurodevelopmental disorders.

Using biochemical fractionation and Western blotting, we describe a decrease in the ratio of GluN2B:GluN2A across the lifespan, from a peak of approximately 1.4:1 in neonates (P7) in the hippocampus. This decrease is consistent with, but more subtle than, calculations of subunit ratios based on EPSC decay times in CA1 of the hippocampus [6]. This difference may arise because of differences in sensitivity between the two methods. It is also possible that GluN2B subunits may be present in a greater percentage of postnatal NMDA receptors than suggested by electrophysiological studies if tri-heterormeric GluN1/GluN2A/GluN2B receptors represent a substantial proportion of receptors in later postnatal life and have faster decay times than assumed during analysis of electrophysiological data [6]. Future studies incorporating electrophysiological measurement of EPSC decay times and ifenprodil sensitivity in discrete brain subregions would be valuable to confirm and extend the findings of this study.

There were a number of limitations of the current study. Firstly, proteins from the PSD were enriched in our samples using a fractionation method based on two rounds of Triton X-100 resuspension/precipitation. This method did not involve use of a sucrose gradient, which is a well-characterized method to separate pre- and parasynaptic proteins from PSD proteins [91, 92]. As such, although presynaptic proteins synaptophysin and Rab3 were scarce in our PSD enrichments (consistent with samples generated using sucrose gradient fractionation [145]), it is likely that non-PSD proteins (particularly presynaptic proteins) were also present in our samples. Secondly, the Western blotting approach used in this study is versatile but is not a gold standard method for protein quantification. We confirmed using loading standard curves that proteins could be quantified effectively. Such standard curves demonstrated that the linear range of quantification for all proteins measured in this study extended sufficiently to allow accurate quantification of at least twofold increases and decreases in samples at the extremes of protein abundance across the lifespan. However, in future work, we will confirm the current findings using another proteomic method, such as mass spectrometry. Thirdly, because traditional loading controls (such as β-actin) are often developmentally regulated, normalization of data to control for loading amount is a challenge. Therefore, we investigated biologically relevant ratios of proteins of interest (such as GluN2B:GluN2A and pY1472-GluN2B:total GluN2B) which are inherently normalized and are also of functional relevance at the synapse. Fourthly, we did not include DTNBP1 heterozygous mice in analysis of NMDA receptor signaling in the hippocampus. Since DTNBP1 heterozygous mice may most closely mimic dysbindin deficits in human psychiatric illness, inclusion of this genotype group in future studies of the hippocampus may expand their relevance to human disease. We also did not detect dysbindin 1A or 1C in our hippocampus PSD enrichments using the Abcam ab133652 antibody. Both proteins have been reported in whole brain mouse PSD enrichments, albeit less abundantly than in synaptosomal membrane fractions [146]. Other antibodies or protein quantification methods may be required to link the developmental trajectory of dysbindin to the emergence of genotype effects in DTNBP1 null mutant mice. Finally, endogenous levels of sex hormones were not measured or controlled for and thus, contributions of sex hormones to sex differences in post-pubertal animals were not examined in this study. Future work employing gonadectomy and hormone replacement may be valuable for determining the extent to which sex differences in NMDA receptor signaling are influenced by sex hormones.

## Conclusions

Taken together, our findings indicate that the GluN2B-GluN2A switch, and associated proteins, are modified in a brain-region-specific fashion by factors which influence risk for neurodevelopmental disorders. Our data suggest that Fyn and PLCγ may be integral to the modulation of the GluN2B-GluN2A switch by sex and DTNBP1 genotype. Future studies investigating the effects of perturbation of this developmental GluN2B-GluN2A switch during critical developmental windows may shed light on whether the imbalance of molecular signaling partners at the synapse disturbs healthy brain function and contributes to risk for neurodevelopmental disorders.

## Additional file

Additional file 1: Supporting information. Figure S1. Synaptophysin abundance in membrane and PSD fractions from the frontal cortex across postnatal development. Figure S2. Representative images of blots used for quantification of NMDA receptor signaling proteins in the hippocampus. Figure S3. Loading standard curves for PSD fractions from the frontal cortex. Figure S4. Loading standard curves for membrane fractions from the frontal cortex. Figure S5. Loading standard curves for PSD fractions from the hippocampus, using P7 samples only. Figure S6. Loading standard curves for PSD fractions from the hippocampus, using P56 samples only. Figure S7. A–G Main effects of age, not described in main text, for proteins quantified in this study; H, I For SRC and PLCγ, distinct developmental trajectories were seen in different subcellular compartments, with stable SRC and PLCγ levels seen in PSD enrichments despite significantly decreasing levels in membranes in general. PSD- postsynaptic density, membranes- crude synaptic membranes (cSM), FCx- frontal cortex, HIP- hippocampus. **p* < 0.05, ***p* < 0.005, ****p* < 0.0005, *****p* < 0.00005, ******p* < 1 × 10^−5^. (DOCX 1627 kb)
