# Supplementary material for: Effects of sex and DTNBP1 (dysbindin) null gene mutation on the developmental GluN2B-GluN2A switch in the mouse cortex and hippocampus
Source: J Neurodev Disord. 2016 May 1;8:14. doi: 10.1186/s11689-016-9148-7 (PMC4852102; doi:10.1186/s11689-016-9148-7)
Supplement: Additional file 1: — Supporting information. Figure S1. Synaptophysin abundance in membrane and PSD fractions from the frontal cortex across postnatal development. Figure S2. Representative images of blots used for quantification of NMDA receptor signaling proteins in the hippocampus. Figure S3. Loading standard curves for PSD fractions from the frontal cortex. Figure S4. Loading standard curves for membrane fractions from the frontal cortex. Figure S5. Loading standard curves for PSD fractions from the hippocampus, using P7 samples only. Figure S6. Loading standard curves for PSD fractions from the hippocampus, using P56 samples only. Figure S7. A–G Main effects of age, not described in main text, for proteins quantified in this study; H, I For SRC and PLCγ, distinct developmental trajectories were seen in different subcellular compartments, with stable SRC and PLCγ levels seen in PSD enrichments despite significantly decreasing levels in membranes in general. PSD- postsynaptic density, membranes- crude synaptic membranes (cSM), FCx- frontal cortex, HIP- hippocampus. *p < 0.05, **p < 0.005, ***p < 0.0005, ****p < 0.00005, *****p < 1 × 10^−5^. (DOCX 1627 kb) [file 11689_2016_9148_MOESM1_ESM.docx]

**Supporting Information**


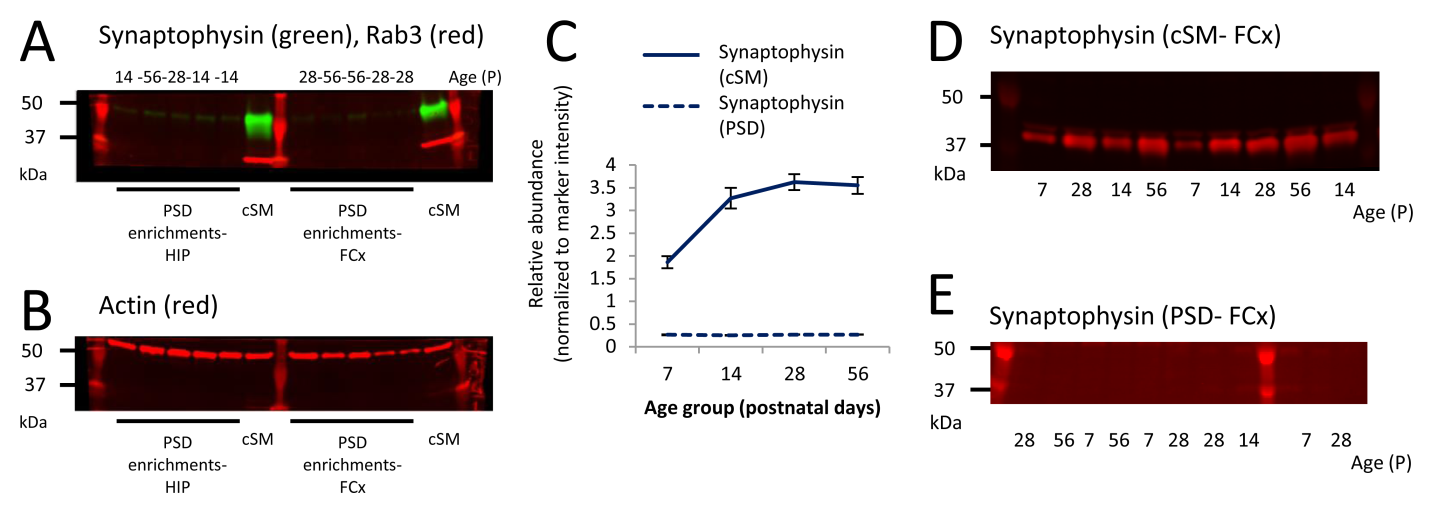


**Supplementary Figure 1. Synaptophysin abundance in membrane and PSD fractions from the frontal cortex across postnatal development**. (A) quantification of levels of synaptophysin and Rab3 in crude membrane preparations and representative PSD enrichments from the main study. Synaptophysin (green) and Rab3 (red) were present at very low levels in PSD enrichments, but were abundant in crude membrane preparations. Loading amounts were the same as those of the main study- for hippocampal PSD enrichments, loading amount= 4ug, frontal cortex PSD enrichments, loading amount= 2ug, crude membrane preparations, loading amount= 10ug; B) in the PSD enrichments from panel A, β-actin was readily detected; C) in the frontal cortex, synaptophysin levels changed dramatically in membrane fractions across development (F(3,36)=17.0, *p*<1x10^-5^), but were not detected in the PSD in the entire cohort; (D) sample western blot of synaptophysin detected in membrane preparations from entire cohort; (E) sample blot of synaptophysin detected in PSD enrichments from entire cohort. PSD- postsynaptic density, kDa- kilodaltons, PSD- postsynaptic density, cSM- crude synaptic membranes, HIP- hippocampus, FCx- frontal cortex, P- postnatal days.


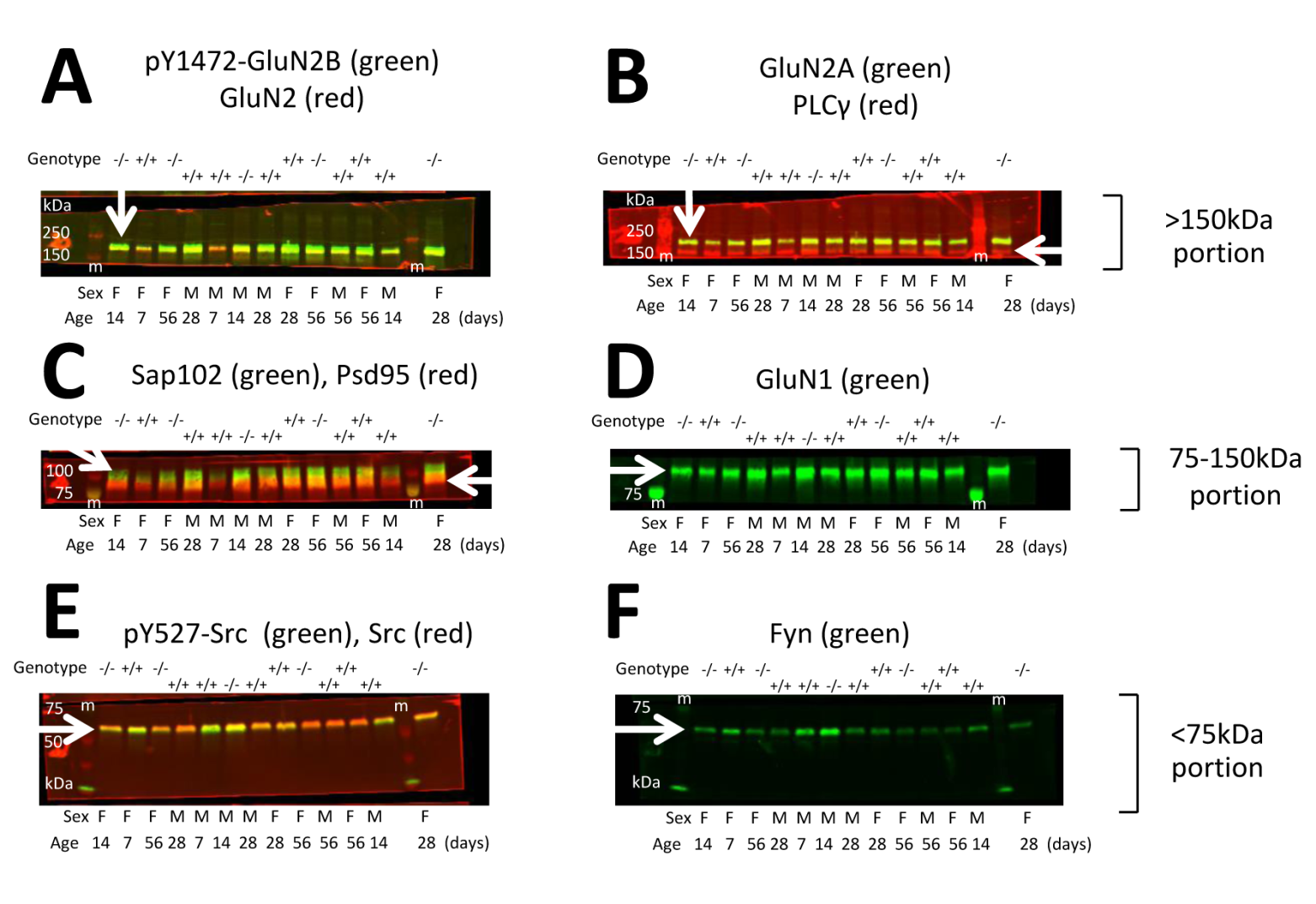


**Supplementary Figure 2. Representative images of blots used for quantification of NMDA receptor signaling proteins in the hippocampus**. For GluN1, GluN2A, GluN2B, PSD-95, SAP102, Src, Fyn, pY1472-GluN2B:total GluN2B and pY527-Src:total Src; 15 lane gels, loading amount= 4ug, blots cut at 75 and 150kDa (white arrows indicate quantified bands). m- marker, kDa- kilodalton, M- male, F- female, -/- homozygyous DTNBP1 null mutant, +/- heterozygous DTNBP null mutant, +/+ wild-type.

**
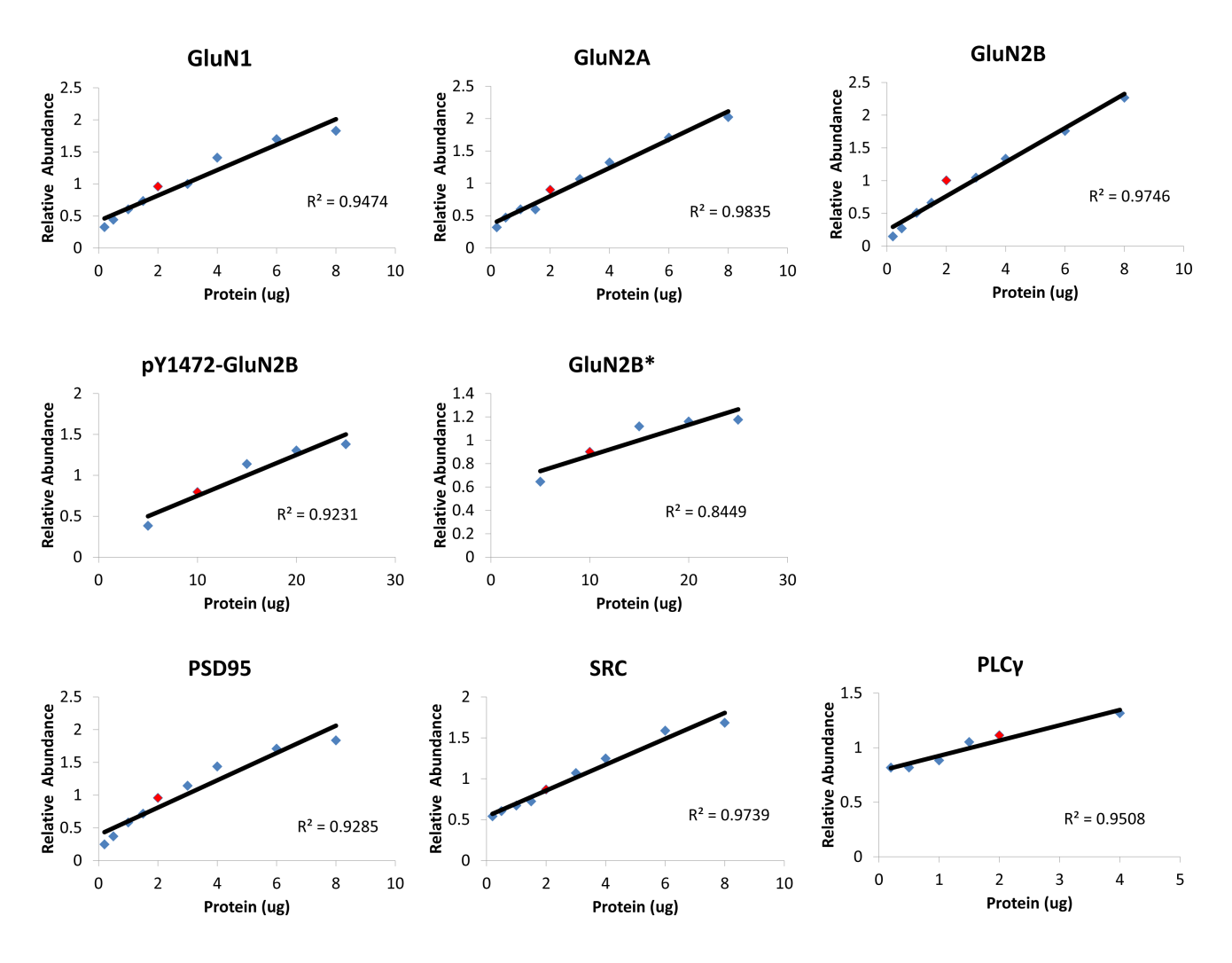
**

**Supplementary Figure 3. Loading standard curves for PSD fractions from the frontal cortex.** The loading amount used for quantification of each protein is indicated on the graphs with red circle. *For quantification of the pY1472 NR2B: total NR2B ratio, 10ug total PSD protein was loaded, while for all other PSD proteins of interest in the frontal cortex, 2ug total PSD protein was loaded. For generating standard curves, a mix of equal microgram amounts from representative P7, 14, 28 and 56 samples was used. P- postnatal days.


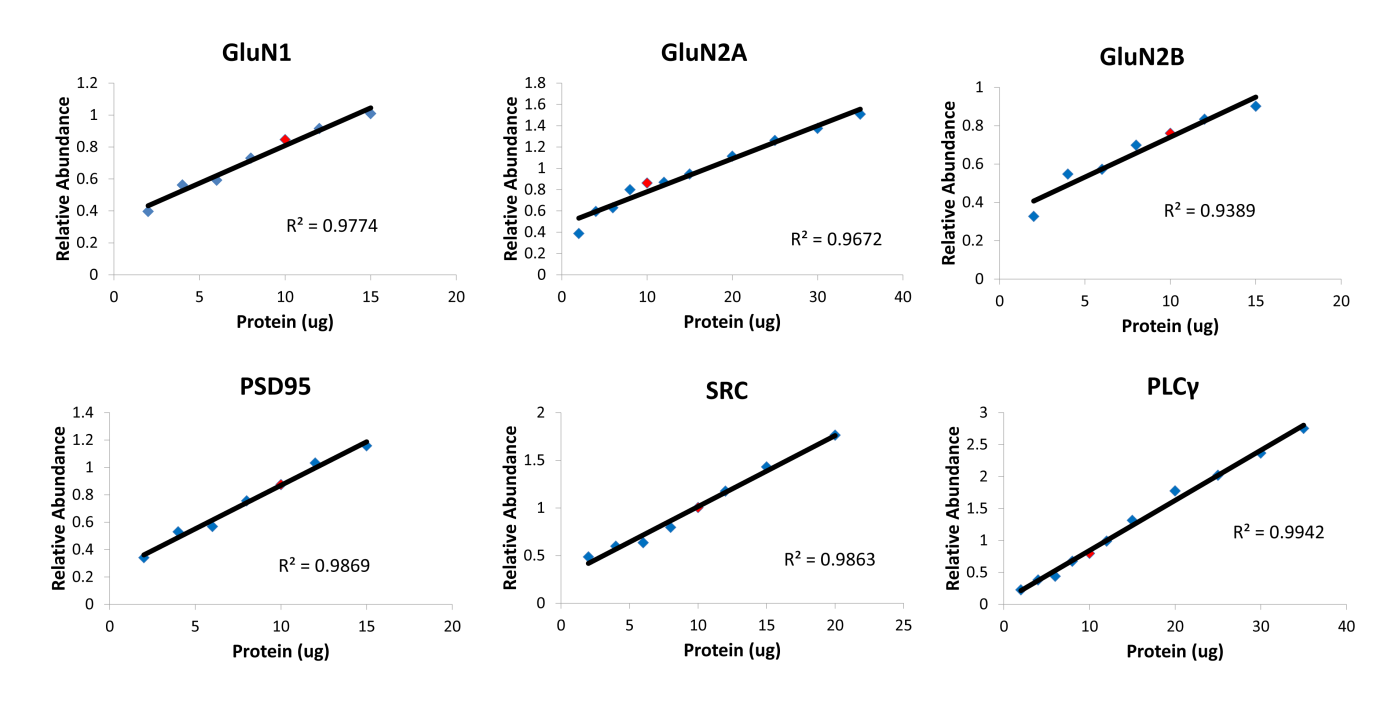
**Supplementary Figure 4. Loading standard curves for membrane fractions from the frontal cortex.** The loading amount used for quantification of each protein is indicated on the graphs with red circle. For generating standard curves, a mix of equal microgram amounts from representative P7, 14, 28 and 56 samples was used.


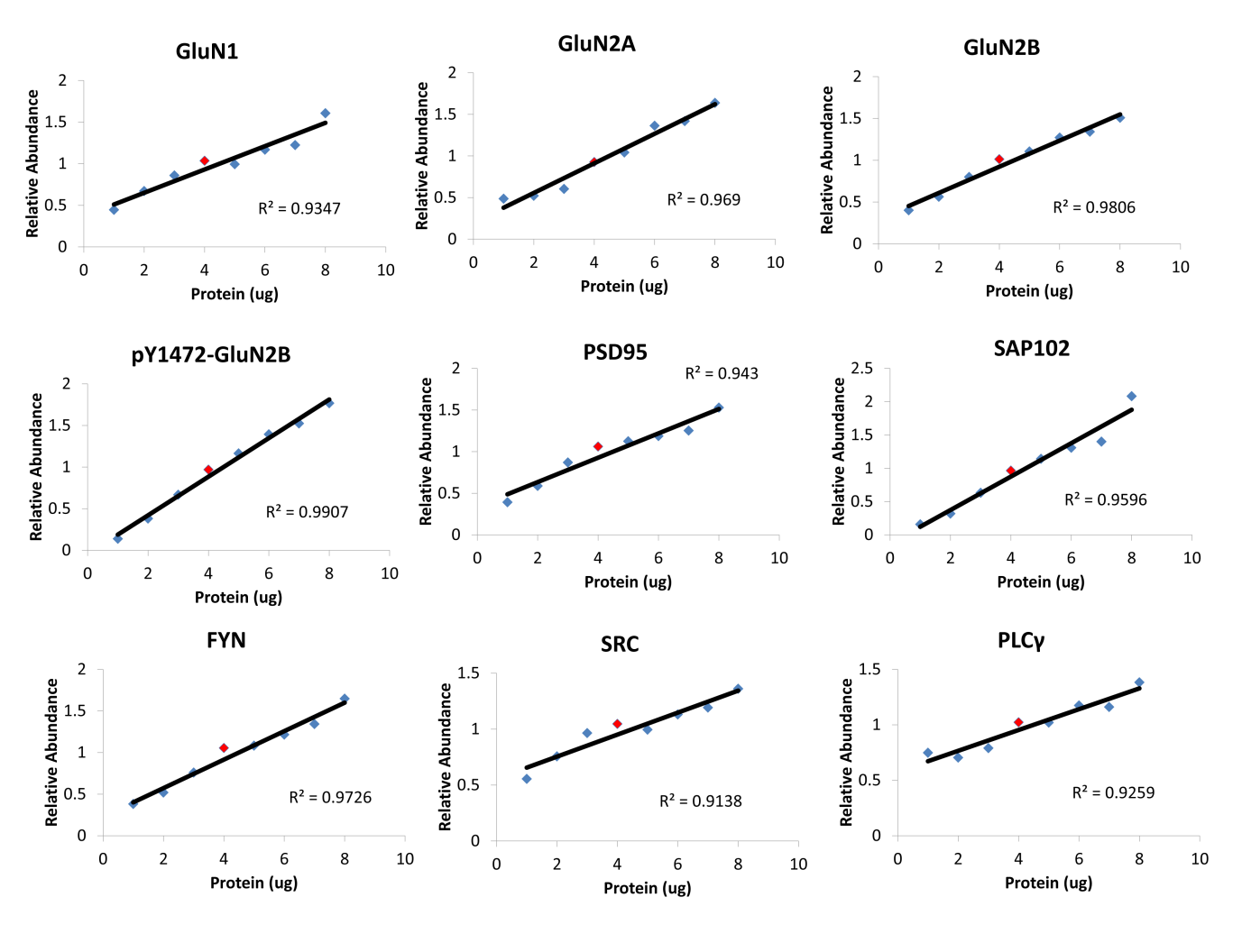
**Supplementary Figure 5. Loading standard curves for PSD fractions from the hippocampus, using P7 samples only.** The loading amount used for quantification of each protein is indicated on the graphs with red circle.


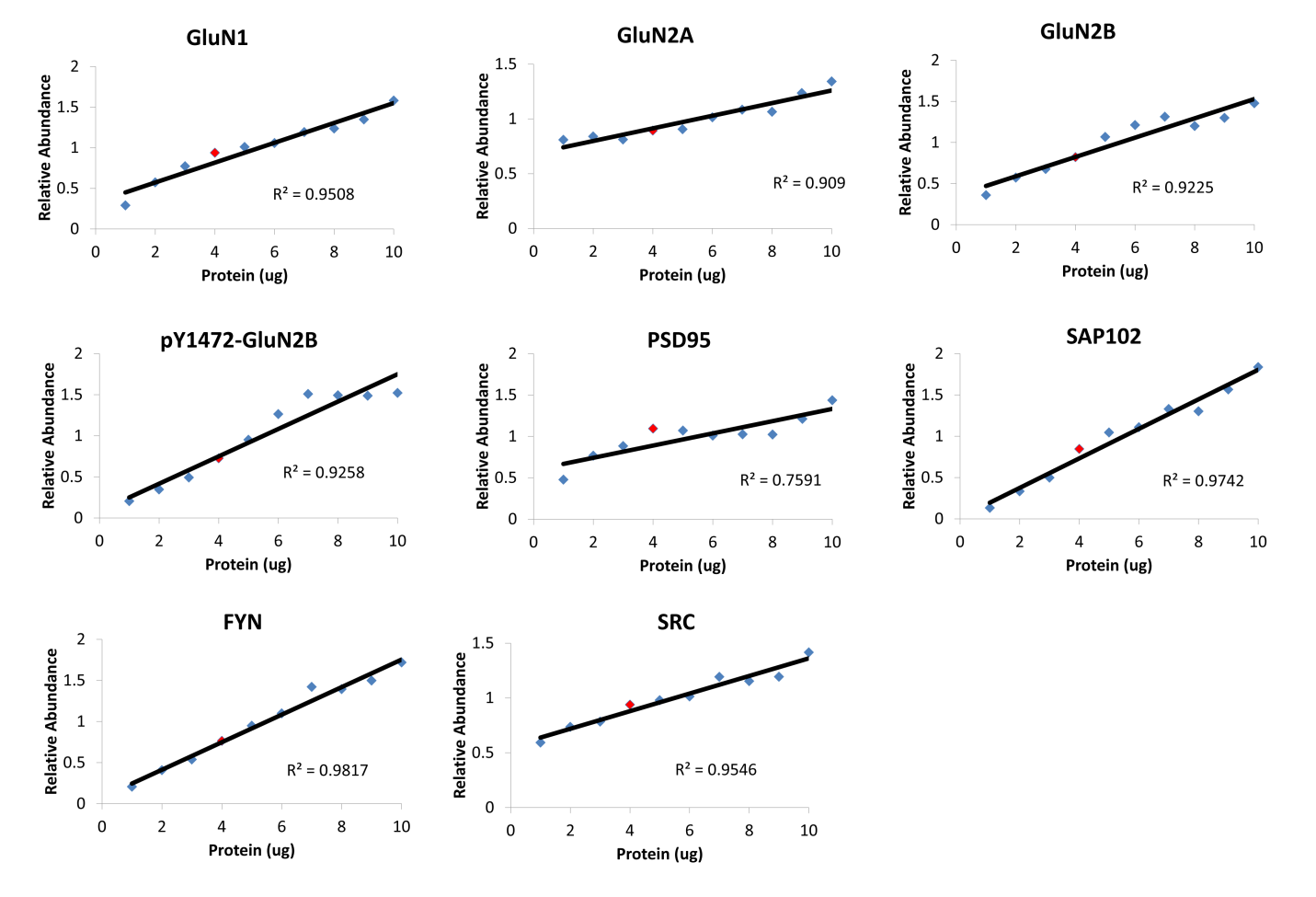
**Supplementary Figure 6. Loading standard curves for PSD fractions from the hippocampus, using P56 samples only.** The loading amount used for quantification of each protein is indicated on the graphs with red circle.


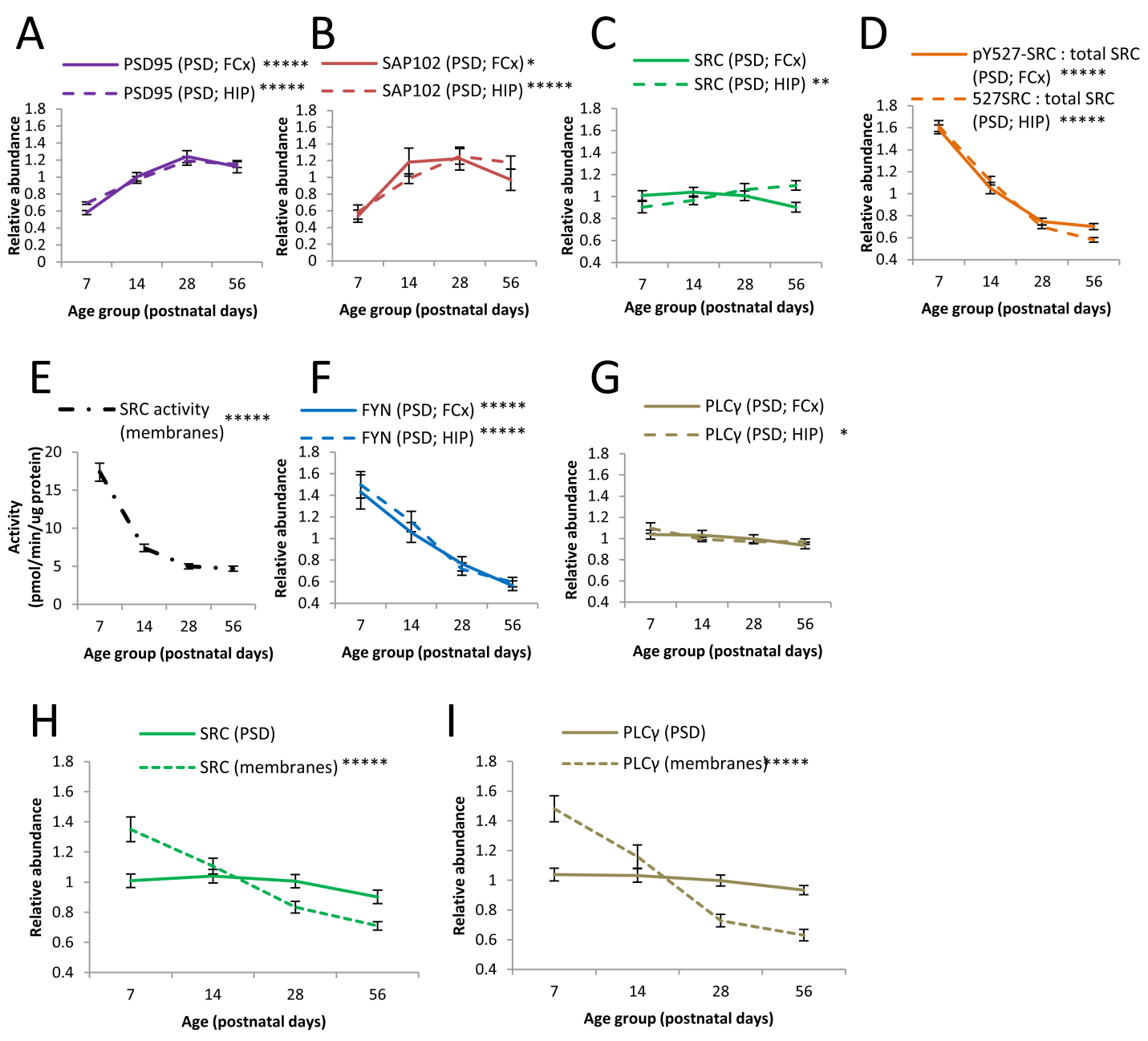


**Supplementary Figure 7.** A-G) Main effects of age, not described in main text, for proteins quantified in this study; H, I) For SRC and PLCγ, distinct developmental trajectories were seen in different subcellular compartments, with stable SRC and PLCγ levels seen in PSD enrichments despite significantly decreasing levels in membranes in general. PSD- postsynaptic density, membranes- crude synaptic membranes (cSM), FCx- frontal cortex, HIP- hippocampus. **p*<0.05, ***p*<0.005, ****p*<0.0005, *****p*<0.00005, ******p*<1x10^-5^.
